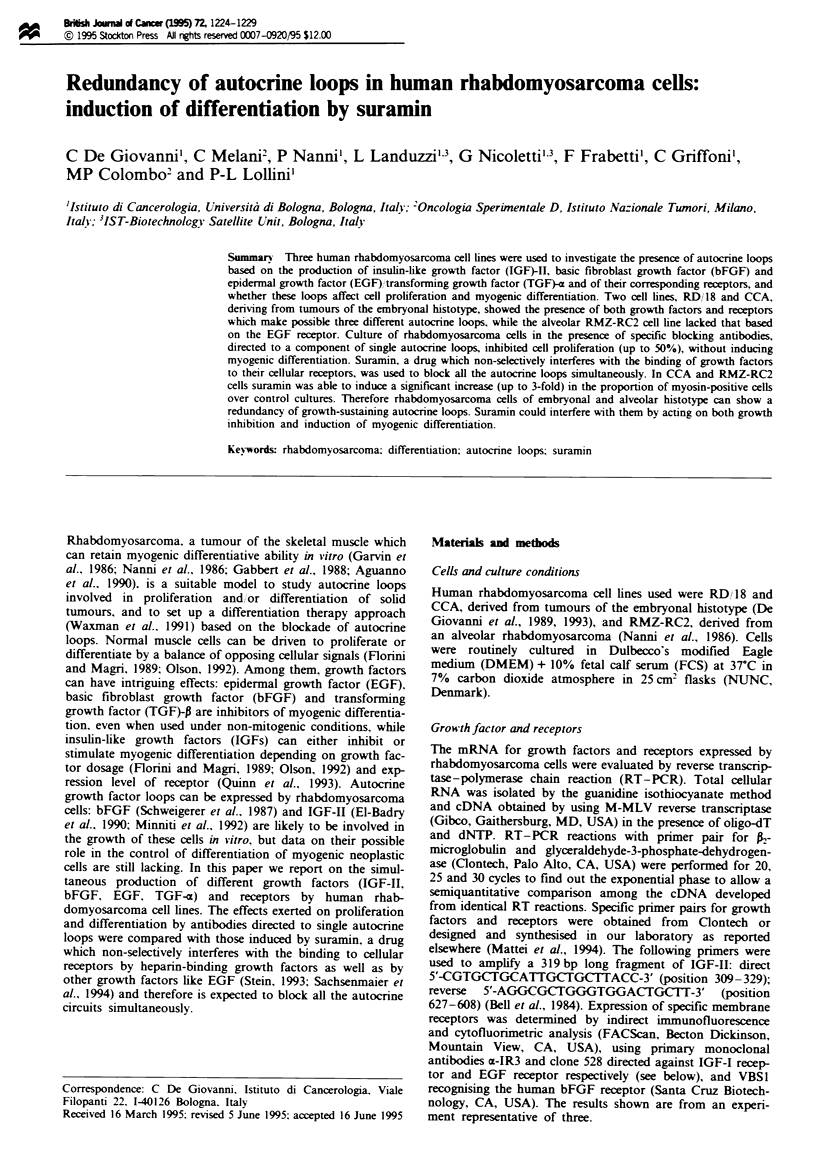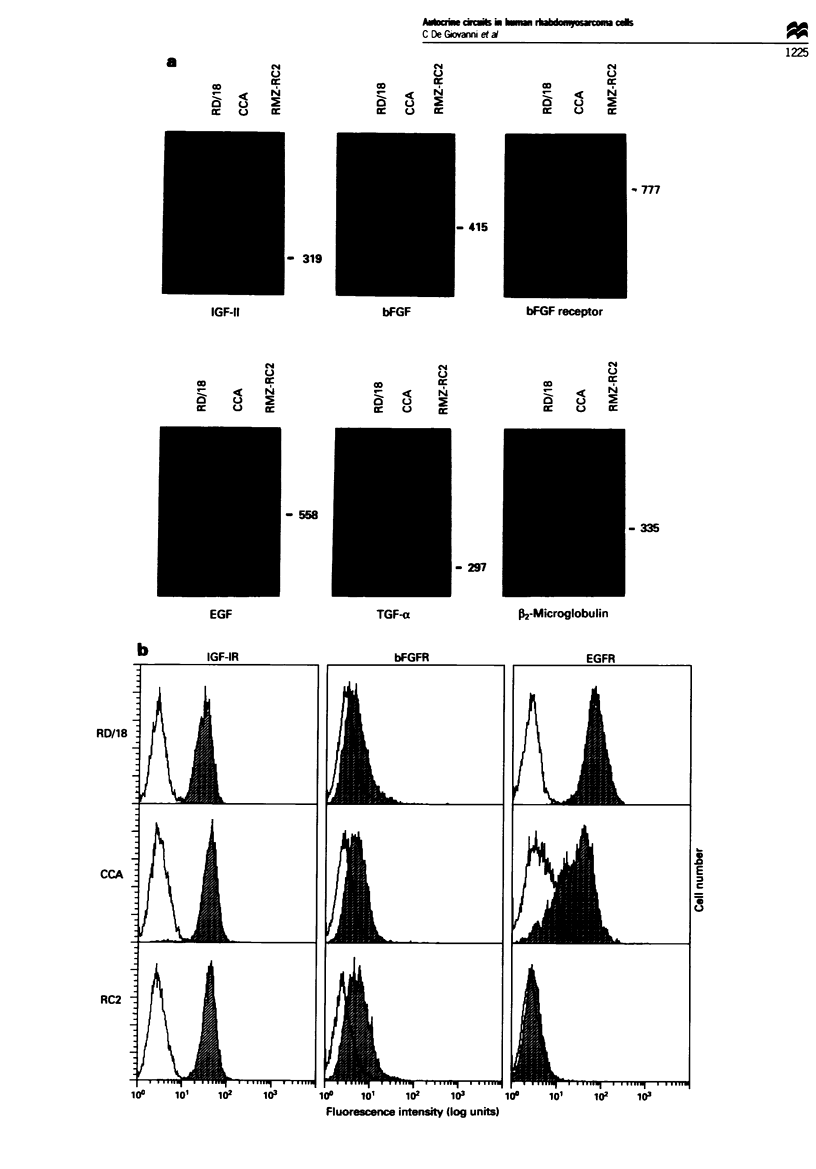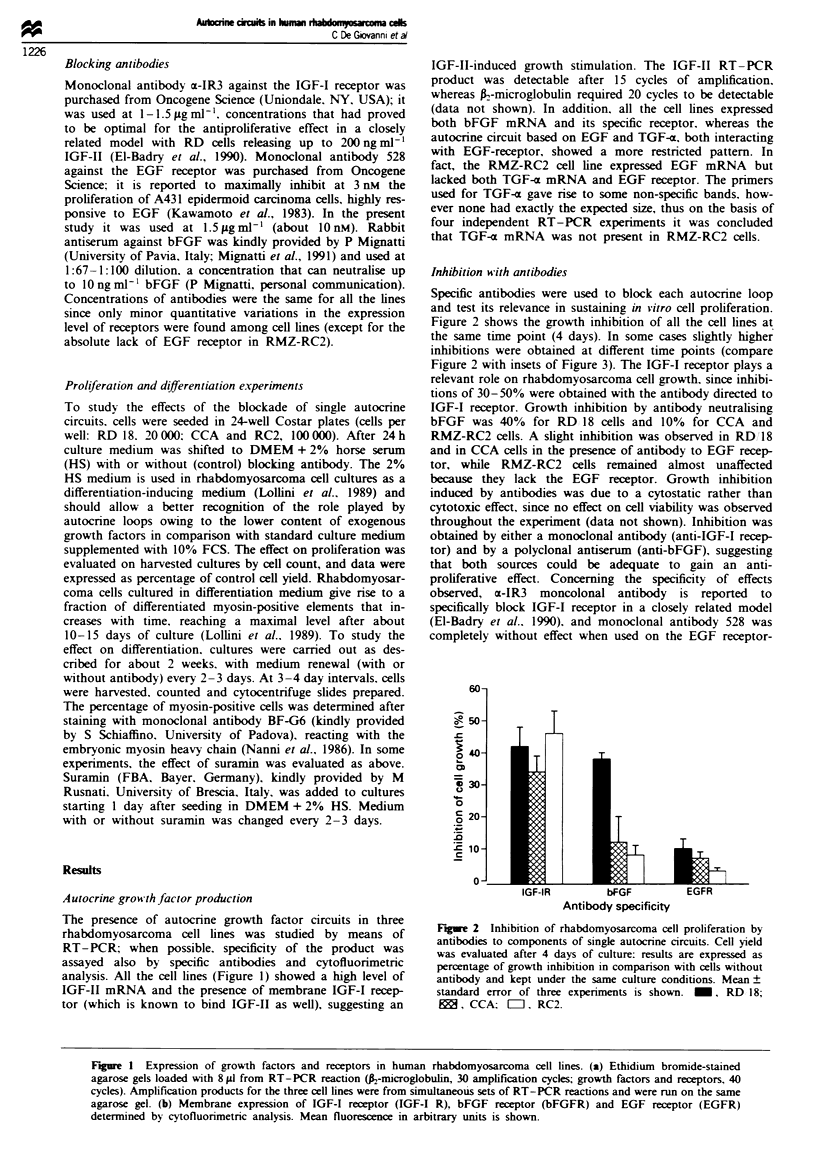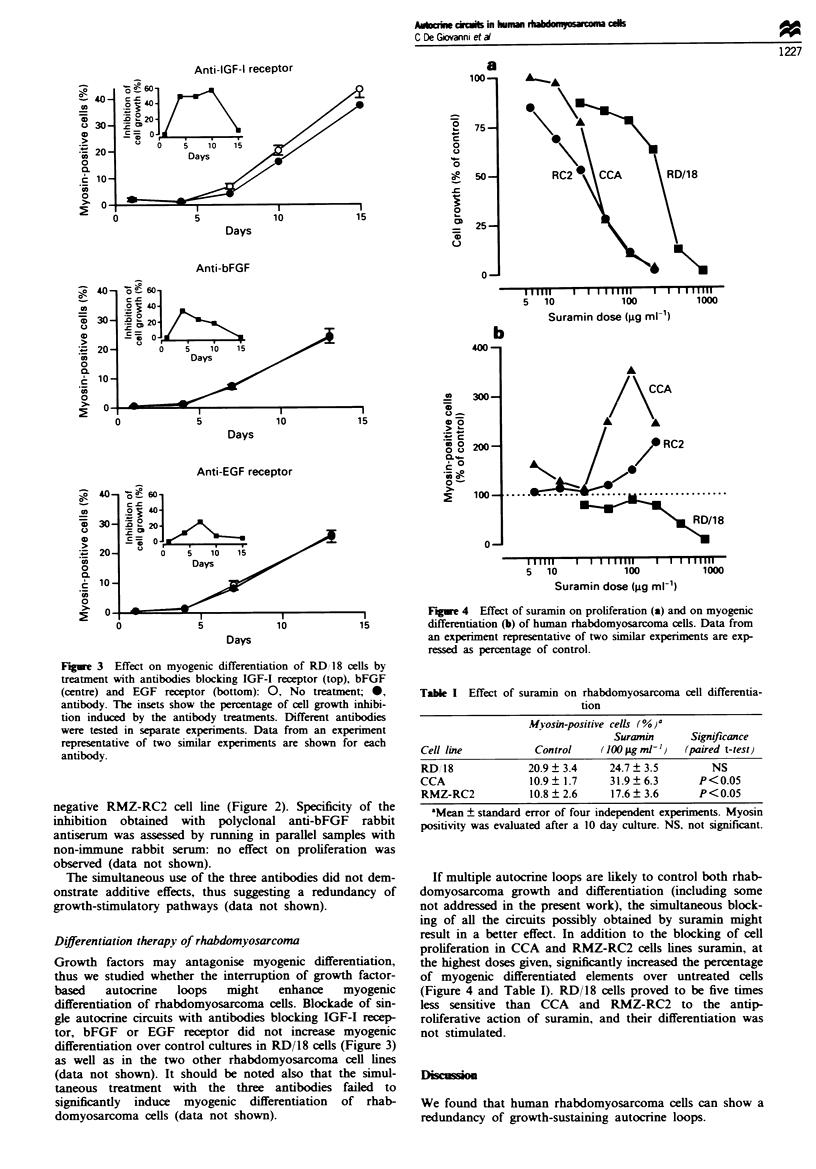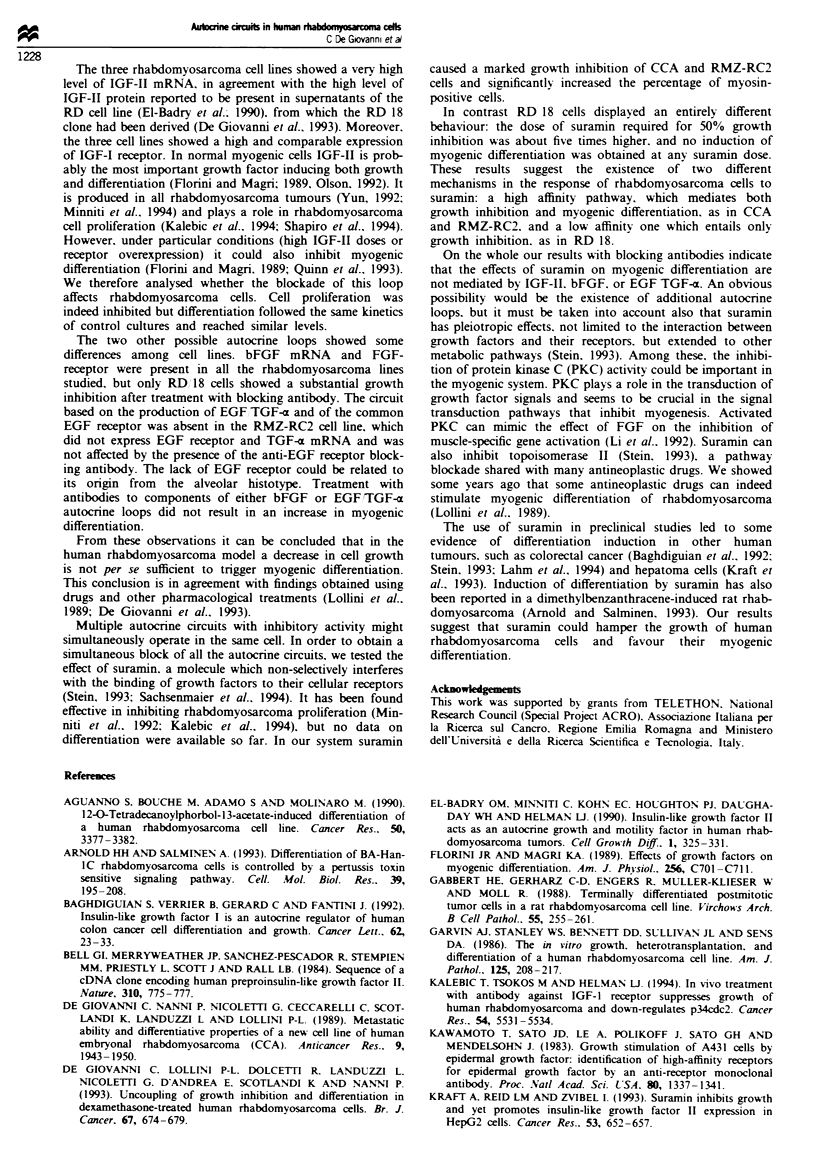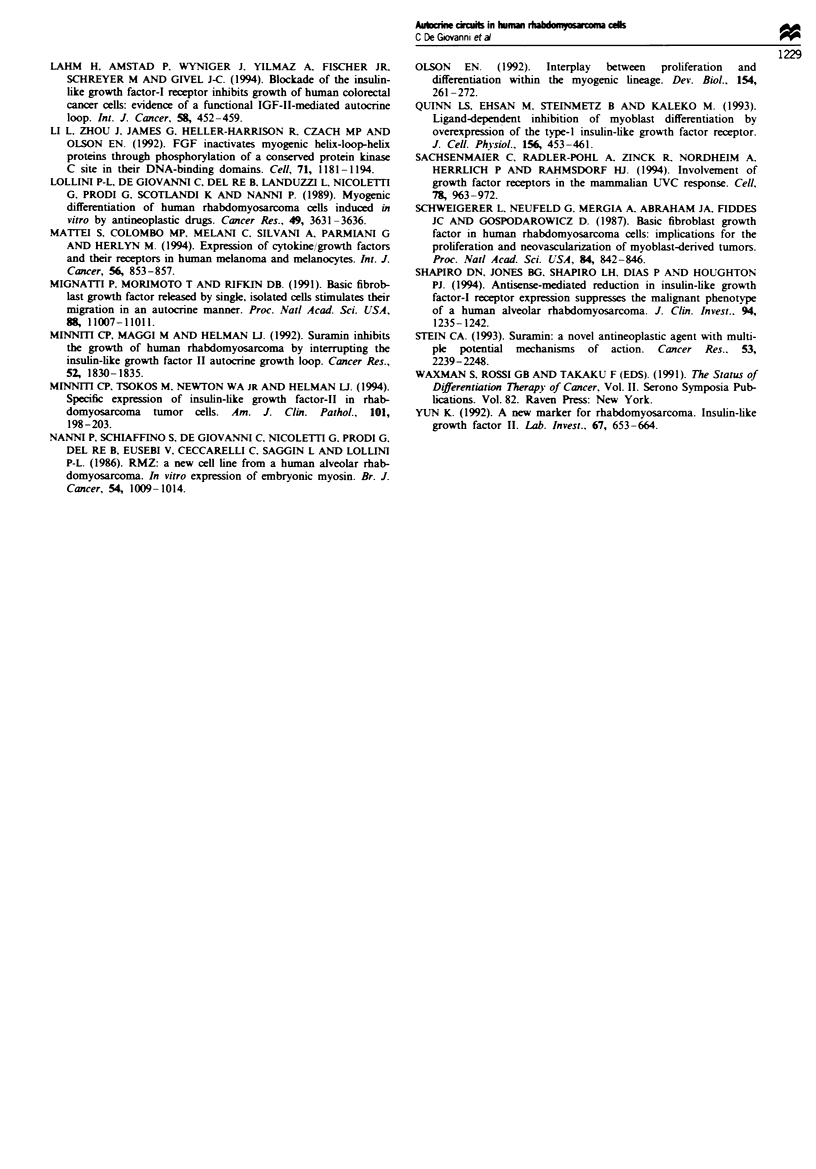# Redundancy of autocrine loops in human rhabdomyosarcoma cells: induction of differentiation by suramin.

**DOI:** 10.1038/bjc.1995.490

**Published:** 1995-11

**Authors:** C. De Giovanni, C. Melani, P. Nanni, L. Landuzzi, G. Nicoletti, F. Frabetti, C. Griffoni, M. P. Colombo, P. L. Lollini

**Affiliations:** Istituto di Cancerologia, Università di Bologna, Italy.

## Abstract

**Images:**


British Journal of Cancer (1995) 72, 1224-1229

© 1995 Stockton Press All rights reserved 0007-0920/95 $12.00

### Redundancy of autocrine loops in human rhabdomyosarcoma cells: induction of differentiation by suramin

C De Giovanni[1], C Melani[2], P Nanni[1], L Landuzzi[1,3], G Nicoletti[1,3], F Frabetti[1], C Griffoni[1], MP Colombo[2] and P-L Lollini[1]

[1]*Istituto di Cancerologia, Università di Bologna, Bologna, Italy;* [2]*Oncologia Sperimentale D, Istituto Nazionale Tumori, Milano, Italy;* [3]*IST-Biotechnology Satellite Unit, Bologna, Italy*

**Summary** Three human rhabdomyosarcoma cell lines were used to investigate the presence of autocrine loops based on the production of insulin-like growth factor (IGF)-II, basic fibroblast growth factor (bFGF) and epidermal growth factor (EGF)/transforming growth factor (TGF)-α and of their corresponding receptors, and whether these loops affect cell proliferation and myogenic differentiation. Two cell lines, RD/18 and CCA, deriving from tumours of the embryonal histotype, showed the presence of both growth factors and receptors which make possible three different autocrine loops, while the alveolar RMZ-RC2 cell line lacked that based on the EGF receptor. Culture of rhabdomyosarcoma cells in the presence of specific blocking antibodies, directed to a component of single autocrine loops, inhibited cell proliferation (up to 50%), without inducing myogenic differentiation. Suramin, a drug which non-selectively interferes with the binding of growth factors to their cellular receptors, was used to block all the autocrine loops simultaneously. In CCA and RMZ-RC2 cells suramin was able to induce a significant increase (up to 3-fold) in the proportion of myosin-positive cells over control cultures. Therefore rhabdomyosarcoma cells of embryonal and alveolar histotype can show a redundancy of growth-sustaining autocrine loops. Suramin could interfere with them by acting on both growth inhibition and induction of myogenic differentiation.

**Keywords:** rhabdomyosarcoma; differentiation; autocrine loops; suramin

Rhabdomyosarcoma, a tumour of the skeletal muscle which can retain myogenic differentiative ability *in vitro* (Garvin *et al.*, 1986; Nanni *et al.*, 1986; Gabbert *et al.*, 1988; Aguanno *et al.*, 1990), is a suitable model to study autocrine loops involved in proliferation and/or differentiation of solid tumours, and to set up a differentiation therapy approach (Waxman *et al.*, 1991) based on the blockade of autocrine loops. Normal muscle cells can be driven to proliferate or differentiate by a balance of opposing cellular signals (Florini and Magri, 1989; Olson, 1992). Among them, growth factors can have intriguing effects: epidermal growth factor (EGF), basic fibroblast growth factor (bFGF) and transforming growth factor (TGF)-β are inhibitors of myogenic differentiation, even when used under non-mitogenic conditions, while insulin-like growth factors (IGFs) can either inhibit or stimulate myogenic differentiation depending on growth factor dosage (Florini and Magri, 1989; Olson, 1992) and expression level of receptor (Quinn *et al.*, 1993). Autocrine growth factor loops can be expressed by rhabdomyosarcoma cells: bFGF (Schweigerer *et al.*, 1987) and IGF-II (El-Badry *et al.*, 1990; Minniti *et al.*, 1992) are likely to be involved in the growth of these cells *in vitro*, but data on their possible role in the control of differentiation of myogenic neoplastic cells are still lacking. In this paper we report on the simultaneous production of different growth factors (IGF-II, bFGF, EGF, TGF-α) and receptors by human rhabdomyosarcoma cell lines. The effects exerted on proliferation and differentiation by antibodies directed to single autocrine loops were compared with those induced by suramin, a drug which non-selectively interferes with the binding to cellular receptors by heparin-binding growth factors as well as by other growth factors like EGF (Stein, 1993; Sachsenmaier *et al.*, 1994) and therefore is expected to block all the autocrine circuits simultaneously.

Correspondence: C De Giovanni, Istituto di Cancerologia, Viale Filopanti 22, I-40126 Bologna, Italy
Received 16 March 1995; revised 5 June 1995; accepted 16 June 1995

### Materials and methods

### *Cells and culture conditions*

Human rhabdomyosarcoma cell lines used were RD/18 and CCA, derived from tumours of the embryonal histotype (De Giovanni *et al.*, 1989, 1993), and RMZ-RC2, derived from an alveolar rhabdomyosarcoma (Nanni *et al.*, 1986). Cells were routinely cultured in Dulbecco's modified Eagle medium (DMEM) + 10% fetal calf serum (FCS) at 37°C in 7% carbon dioxide atmosphere in 25 cm$^2$ flasks (NUNC, Denmark).

### *Growth factor and receptors*

The mRNA for growth factors and receptors expressed by rhabdomyosarcoma cells were evaluated by reverse transcriptase-polymerase chain reaction (RT-PCR). Total cellular RNA was isolated by the guanidine isothiocyanate method and cDNA obtained by using M-MLV reverse transcriptase (Gibco, Gaithersburg, MD, USA) in the presence of oligo-dT and dNTP. RT-PCR reactions with primer pair for $\beta_2$-microglobulin and glyceraldehyde-3-phosphate-dehydrogenase (Clontech, Palo Alto, CA, USA) were performed for 20, 25 and 30 cycles to find out the exponential phase to allow a semiquantitative comparison among the cDNA developed from identical RT reactions. Specific primer pairs for growth factors and receptors were obtained from Clontech or designed and synthesised in our laboratory as reported elsewhere (Mattei *et al.*, 1994). The following primers were used to amplify a 319 bp long fragment of IGF-II: direct 5'-CGTGCTGCATTGCTGCTTACC-3' (position 309-329); reverse 5'-AGGCGCTGGGTGGACTGCTT-3' (position 627-608) (Bell *et al.*, 1984). Expression of specific membrane receptors was determined by indirect immunofluorescence and cytofluorimetric analysis (FACScan, Becton Dickinson, Mountain View, CA, USA), using primary monoclonal antibodies α-IR3 and clone 528 directed against IGF-I receptor and EGF receptor respectively (see below), and VBS1 recognising the human bFGF receptor (Santa Cruz Biotechnology, CA, USA). The results shown are from an experiment representative of three.

a

RD/18

CCA

RMZ-RC2

319

415

777

IGF-II

bFGF

bFGF receptor

558

297

335

EGF

TGF-α

β2-Microglobulin b

IGF-IR

bFGFR

EGFR

RD/18

CCA

RC2

Cell number

Fluorescence intensity (log units)

*Blocking antibodies*

Monoclonal antibody α-IR3 against the IGF-I receptor was purchased from Oncogene Science (Uniondale, NY, USA); it was used at 1–1.5 μg $ml^{-1}$, concentrations that had proved to be optimal for the antiproliferative effect in a closely related model with RD cells releasing up to 200 ng $ml^{-1}$ IGF-II (El-Badry *et al.*, 1990). Monoclonal antibody 528 against the EGF receptor was purchased from Oncogene Science; it is reported to maximally inhibit at 3 nM the proliferation of A431 epidermoid carcinoma cells, highly responsive to EGF (Kawamoto *et al.*, 1983). In the present study it was used at 1.5 μg $ml^{-1}$ (about 10 nM). Rabbit antiserum against bFGF was kindly provided by P Mignatti (University of Pavia, Italy; Mignatti *et al.*, 1991) and used at 1:67–1:100 dilution, a concentration that can neutralise up to 10 ng $ml^{-1}$ bFGF (P Mignatti, personal communication). Concentrations of antibodies were the same for all the lines since only minor quantitative variations in the expression level of receptors were found among cell lines (except for the absolute lack of EGF receptor in RMZ-RC2).

*Proliferation and differentiation experiments*

To study the effects of the blockade of single autocrine circuits, cells were seeded in 24-well Costar plates (cells per well: RD 18, 20 000; CCA and RC2, 100 000). After 24 h culture medium was shifted to DMEM + 2% horse serum (HS) with or without (control) blocking antibody. The 2% HS medium is used in rhabdomyosarcoma cell cultures as a differentiation-inducing medium (Lollini *et al.*, 1989) and should allow a better recognition of the role played by autocrine loops owing to the lower content of exogenous growth factors in comparison with standard culture medium supplemented with 10% FCS. The effect on proliferation was evaluated on harvested cultures by cell count, and data were expressed as percentage of control cell yield. Rhabdomyosarcoma cells cultured in differentiation medium give rise to a fraction of differentiated myosin-positive elements that increases with time, reaching a maximal level after about 10–15 days of culture (Lollini *et al.*, 1989). To study the effect on differentiation, cultures were carried out as described for about 2 weeks, with medium renewal (with or without antibody) every 2–3 days. At 3–4 day intervals, cells were harvested, counted and cytocentrifuge slides prepared. The percentage of myosin-positive cells was determined after staining with monoclonal antibody BF-G6 (kindly provided by S Schiaffino, University of Padova), reacting with the embryonic myosin heavy chain (Nanni *et al.*, 1986). In some experiments, the effect of suramin was evaluated as above. Suramin (FBA, Bayer, Germany), kindly provided by M Rusnati, University of Brescia, Italy, was added to cultures starting 1 day after seeding in DMEM + 2% HS. Medium with or without suramin was changed every 2–3 days.

### Results

*Autocrine growth factor production*

The presence of autocrine growth factor circuits in three rhabdomyosarcoma cell lines was studied by means of RT–PCR; when possible, specificity of the product was assayed also by specific antibodies and cytofluorimetric analysis. All the cell lines (Figure 1) showed a high level of IGF-II mRNA and the presence of membrane IGF-I receptor (which is known to bind IGF-II as well), suggesting an IGF-II-induced growth stimulation. The IGF-II RT–PCR product was detectable after 15 cycles of amplification, whereas $\beta_2$-microglobulin required 20 cycles to be detectable (data not shown). In addition, all the cell lines expressed both bFGF mRNA and its specific receptor, whereas the autocrine circuit based on EGF and TGF-α, both interacting with EGF-receptor, showed a more restricted pattern. In fact, the RMZ-RC2 cell line expressed EGF mRNA but lacked both TGF-α mRNA and EGF receptor. The primers used for TGF-α gave rise to some non-specific bands, however none had exactly the expected size, thus on the basis of four independent RT–PCR experiments it was concluded that TGF-α mRNA was not present in RMZ-RC2 cells.

*Inhibition with antibodies*

Specific antibodies were used to block each autocrine loop and test its relevance in sustaining *in vitro* cell proliferation. Figure 2 shows the growth inhibition of all the cell lines at the same time point (4 days). In some cases slightly higher inhibitions were obtained at different time points (compare Figure 2 with insets of Figure 3). The IGF-I receptor plays a relevant role on rhabdomyosarcoma cell growth, since inhibitions of 30–50% were obtained with the antibody directed to IGF-I receptor. Growth inhibition by antibody neutralising bFGF was 40% for RD/18 cells and 10% for CCA and RMZ-RC2 cells. A slight inhibition was observed in RD/18 and in CCA cells in the presence of antibody to EGF receptor, while RMZ-RC2 cells remained almost unaffected because they lack the EGF receptor. Growth inhibition induced by antibodies was due to a cytostatic rather than cytotoxic effect, since no effect on cell viability was observed throughout the experiment (data not shown). Inhibition was obtained by either a monoclonal antibody (anti-IGF-I receptor) and by a polyclonal antiserum (anti-bFGF), suggesting that both sources could be adequate to gain an antiproliferative effect. Concerning the specificity of effects observed, α-IR3 moncolonal antibody is reported to specifically block IGF-I receptor in a closely related model (El-Badry *et al.*, 1990), and monoclonal antibody 528 was completely without effect when used on the EGF receptor-

**Figure 2** Inhibition of rhabdomyosarcoma cell proliferation by antibodies to components of single autocrine circuits. Cell yield was evaluated after 4 days of culture; results are expressed as percentage of growth inhibition in comparison with cells without antibody and kept under the same culture conditions. Mean ± standard error of three experiments is shown. ■, RD/18; ▩, CCA; □, RC2.

**Figure 1** Expression of growth factors and receptors in human rhabdomyosarcoma cell lines. (**a**) Ethidium bromide-stained agarose gels loaded with 8 μl from RT–PCR reaction ($\beta_2$-microglobulin, 30 amplification cycles; growth factors and receptors, 40 cycles). Amplification products for the three cell lines were from simultaneous sets of RT–PCR reactions and were run on the same agarose gel. (**b**) Membrane expression of IGF-I receptor (IGF-I R), bFGF receptor (bFGFR) and EGF receptor (EGFR) determined by cytofluorimetric analysis. Mean fluorescence in arbitrary units is shown.

**Figure 3** Effect on myogenic differentiation of RD/18 cells by treatment with antibodies blocking IGF-I receptor (top), bFGF (centre) and EGF receptor (bottom): O, No treatment; ●, antibody. The insets show the percentage of cell growth inhibition induced by the antibody treatments. Different antibodies were tested in separate experiments. Data from an experiment representative of two similar experiments are shown for each antibody.

negative RMZ-RC2 cell line (Figure 2). Specificity of the inhibition obtained with polyclonal anti-bFGF rabbit antiserum was assessed by running in parallel samples with non-immune rabbit serum: no effect on proliferation was observed (data not shown).

The simultaneous use of the three antibodies did not demonstrate additive effects, thus suggesting a redundancy of growth-stimulatory pathways (data not shown).

### *Differentiation therapy of rhabdomyosarcoma*

Growth factors may antagonise myogenic differentiation, thus we studied whether the interruption of growth factor-based autocrine loops might enhance myogenic differentiation of rhabdomyosarcoma cells. Blockade of single autocrine circuits with antibodies blocking IGF-I receptor, bFGF or EGF receptor did not increase myogenic differentiation over control cultures in RD/18 cells (Figure 3) as well as in the two other rhabdomyosarcoma cell lines (data not shown). It should be noted also that the simultaneous treatment with the three antibodies failed to significantly induce myogenic differentiation of rhabdomyosarcoma cells (data not shown).

**Figure 4** Effect of suramin on proliferation (**a**) and on myogenic differentiation (**b**) of human rhabdomyosarcoma cells. Data from an experiment representative of two similar experiments are expressed as percentage of control.

**Table I** Effect of suramin on rhabdomyosarcoma cell differentiation

| | Myosin-positive cells (%)[a] | | |
|---|---|---|---|
| *Cell line* | *Control* | *Suramin (100 µg ml⁻¹)* | *Significance (paired* t*-test)* |
| RD/18 | 20.9 ± 3.4 | 24.7 ± 3.5 | NS |
| CCA | 10.9 ± 1.7 | 31.9 ± 6.3 | $P<0.05$ |
| RMZ-RC2 | 10.8 ± 2.6 | 17.6 ± 3.6 | $P<0.05$ |

[a]Mean ± standard error of four independent experiments. Myosin positivity was evaluated after a 10 day culture. NS, not significant.

If multiple autocrine loops are likely to control both rhabdomyosarcoma growth and differentiation (including some not addressed in the present work), the simultaneous blocking of all the circuits possibly obtained by suramin might result in a better effect. In addition to the blocking of cell proliferation in CCA and RMZ-RC2 cells lines suramin, at the highest doses given, significantly increased the percentage of myogenic differentiated elements over untreated cells (Figure 4 and Table I). RD/18 cells proved to be five times less sensitive than CCA and RMZ-RC2 to the antiproliferative action of suramin, and their differentiation was not stimulated.

### Discussion

We found that human rhabdomyosarcoma cells can show a redundancy of growth-sustaining autocrine loops.

The three rhabdomyosarcoma cell lines showed a very high level of IGF-II mRNA, in agreement with the high level of IGF-II protein reported to be present in supernatants of the RD cell line (El-Badry *et al.*, 1990), from which the RD 18 clone had been derived (De Giovanni *et al.*, 1993). Moreover, the three cell lines showed a high and comparable expression of IGF-I receptor. In normal myogenic cells IGF-II is probably the most important growth factor inducing both growth and differentiation (Florini and Magri; 1989, Olson, 1992). It is produced in all rhabdomyosarcoma tumours (Yun, 1992; Minniti *et al.*, 1994) and plays a role in rhabdomyosarcoma cell proliferation (Kalebic *et al.*, 1994; Shapiro *et al.*, 1994). However, under particular conditions (high IGF-II doses or receptor overexpression) it could also inhibit myogenic differentiation (Florini and Magri, 1989; Quinn *et al.*, 1993). We therefore analysed whether the blockade of this loop affects rhabdomyosarcoma cells. Cell proliferation was indeed inhibited but differentiation followed the same kinetics of control cultures and reached similar levels.

The two other possible autocrine loops showed some differences among cell lines. bFGF mRNA and FGF-receptor were present in all the rhabdomyosarcoma lines studied, but only RD/18 cells showed a substantial growth inhibition after treatment with blocking antibody. The circuit based on the production of EGF/TGF-α and of the common EGF receptor was absent in the RMZ-RC2 cell line, which did not express EGF receptor and TGF-α mRNA and was not affected by the presence of the anti-EGF receptor blocking antibody. The lack of EGF receptor could be related to its origin from the alveolar histotype. Treatment with antibodies to components of either bFGF or EGF/TGF-α autocrine loops did not result in an increase in myogenic differentiation.

From these observations it can be concluded that in the human rhabdomyosarcoma model a decrease in cell growth is not *per se* sufficient to trigger myogenic differentiation. This conclusion is in agreement with findings obtained using drugs and other pharmacological treatments (Lollini *et al.*, 1989; De Giovanni *et al.*, 1993).

Multiple autocrine circuits with inhibitory activity might simultaneously operate in the same cell. In order to obtain a simultaneous block of all the autocrine circuits, we tested the effect of suramin, a molecule which non-selectively interferes with the binding of growth factors to their cellular receptors (Stein, 1993; Sachsenmaier *et al.*, 1994). It has been found effective in inhibiting rhabdomyosarcoma proliferation (Minniti *et al.*, 1992; Kalebic *et al.*, 1994), but no data on differentiation were available so far. In our system suramin caused a marked growth inhibition of CCA and RMZ-RC2 cells and significantly increased the percentage of myosin-positive cells.

In contrast RD 18 cells displayed an entirely different behaviour: the dose of suramin required for 50% growth inhibition was about five times higher, and no induction of myogenic differentiation was obtained at any suramin dose. These results suggest the existence of two different mechanisms in the response of rhabdomyosarcoma cells to suramin: a high affinity pathway, which mediates both growth inhibition and myogenic differentiation, as in CCA and RMZ-RC2, and a low affinity one which entails only growth inhibition, as in RD 18.

On the whole our results with blocking antibodies indicate that the effects of suramin on myogenic differentiation are not mediated by IGF-II, bFGF, or EGF/TGF-α. An obvious possibility would be the existence of additional autocrine loops, but it must be taken into account also that suramin has pleiotropic effects, not limited to the interaction between growth factors and their receptors, but extended to other metabolic pathways (Stein, 1993). Among these, the inhibition of protein kinase C (PKC) activity could be important in the myogenic system. PKC plays a role in the transduction of growth factor signals and seems to be crucial in the signal transduction pathways that inhibit myogenesis. Activated PKC can mimic the effect of FGF on the inhibition of muscle-specific gene activation (Li *et al.*, 1992). Suramin can also inhibit topoisomerase II (Stein, 1993), a pathway blockade shared with many antineoplastic drugs. We showed some years ago that some antineoplastic drugs can indeed stimulate myogenic differentiation of rhabdomyosarcoma (Lollini *et al.*, 1989).

The use of suramin in preclinical studies led to some evidence of differentiation induction in other human tumours, such as colorectal cancer (Baghdiguian *et al.*, 1992; Stein, 1993; Lahm *et al.*, 1994) and hepatoma cells (Kraft *et al.*, 1993). Induction of differentiation by suramin has also been reported in a dimethylbenzanthracene-induced rat rhabdomyosarcoma (Arnold and Salminen, 1993). Our results suggest that suramin could hamper the growth of human rhabdomyosarcoma cells and favour their myogenic differentiation.

**Acknowledgements**
This work was supported by grants from TELETHON, National Research Council (Special Project ACRO), Associazione Italiana per la Ricerca sul Cancro, Regione Emilia Romagna and Ministero dell'Università e della Ricerca Scientifica e Tecnologia, Italy.

**References**

AGUANNO S, BOUCHE M, ADAMO S AND MOLINARO M. (1990). 12-O-Tetradecanoylphorbol-13-acetate-induced differentiation of a human rhabdomyosarcoma cell line. *Cancer Res.*, **50**, 3377-3382.

ARNOLD HH AND SALMINEN A. (1993). Differentiation of BA-Han-1C rhabdomyosarcoma cells is controlled by a pertussis toxin sensitive signaling pathway. *Cell. Mol. Biol. Res.*, **39**, 195-208.

BAGHDIGUIAN S, VERRIER B, GERARD C AND FANTINI J. (1992). Insulin-like growth factor I is an autocrine regulator of human colon cancer cell differentiation and growth. *Cancer Lett.*, **62**, 23-33.

BELL GI, MERRYWEATHER JP, SANCHEZ-PESCADOR R, STEMPIEN MM, PRIESTLY L, SCOTT J AND RALL LB. (1984). Sequence of a cDNA clone encoding human preproinsulin-like growth factor II. *Nature*, **310**, 775-777.

DE GIOVANNI C, NANNI P, NICOLETTI G, CECCARELLI C, SCOTLANDI K, LANDUZZI L AND LOLLINI P-L. (1989). Metastatic ability and differentiative properties of a new cell line of human embryonal rhabdomyosarcoma (CCA). *Anticancer Res.*, **9**, 1943-1950.

DE GIOVANNI C, LOLLINI P-L, DOLCETTI R, LANDUZZI L, NICOLETTI G, D'ANDREA E, SCOTLANDI K AND NANNI P. (1993). Uncoupling of growth inhibition and differentiation in dexamethasone-treated human rhabdomyosarcoma cells. *Br. J. Cancer*, **67**, 674-679.

EL-BADRY OM, MINNITI C, KOHN EC, HOUGHTON PJ, DAUGHADAY WH AND HELMAN LJ. (1990). Insulin-like growth factor II acts as an autocrine growth and motility factor in human rhabdomyosarcoma tumors. *Cell Growth Diff.*, **1**, 325-331.

FLORINI JR AND MAGRI KA. (1989). Effects of growth factors on myogenic differentiation. *Am. J. Physiol.*, **256**, C701-C711.

GABBERT HE, GERHARZ C-D, ENGERS R, MULLER-KLIESER W AND MOLL R. (1988). Terminally differentiated postmitotic tumor cells in a rat rhabdomyosarcoma cell line. *Virchows Arch. B Cell Pathol.*, **55**, 255-261.

GARVIN AJ, STANLEY WS, BENNETT DD, SULLIVAN JL AND SENS DA. (1986). The *in vitro* growth, heterotransplantation, and differentiation of a human rhabdomyosarcoma cell line. *Am. J. Pathol.*, **125**, 208-217.

KALEBIC T, TSOKOS M AND HELMAN LJ. (1994). In vivo treatment with antibody against IGF-1 receptor suppresses growth of human rhabdomyosarcoma and down-regulates p34cdc2. *Cancer Res.*, **54**, 5531-5534.

KAWAMOTO T, SATO JD, LE A, POLIKOFF J, SATO GH AND MENDELSOHN J. (1983). Growth stimulation of A431 cells by epidermal growth factor: identification of high-affinity receptors for epidermal growth factor by an anti-receptor monoclonal antibody. *Proc. Natl Acad. Sci. USA*, **80**, 1337-1341.

KRAFT A, REID LM AND ZVIBEL I. (1993). Suramin inhibits growth and yet promotes insulin-like growth factor II expression in HepG2 cells. *Cancer Res.*, **53**, 652-657.

LAHM H, AMSTAD P, WYNIGER J, YILMAZ A, FISCHER JR, SCHREYER M AND GIVEL J-C. (1994). Blockade of the insulin-like growth factor-I receptor inhibits growth of human colorectal cancer cells: evidence of a functional IGF-II-mediated autocrine loop. *Int. J. Cancer*, **58**, 452-459.

LI L, ZHOU J, JAMES G, HELLER-HARRISON R, CZACH MP AND OLSON EN. (1992). FGF inactivates myogenic helix-loop-helix proteins through phosphorylation of a conserved protein kinase C site in their DNA-binding domains. *Cell*, **71**, 1181-1194.

LOLLINI P-L, DE GIOVANNI C, DEL RE B, LANDUZZI L, NICOLETTI G, PRODI G, SCOTLANDI K AND NANNI P. (1989). Myogenic differentiation of human rhabdomyosarcoma cells induced *in vitro* by antineoplastic drugs. *Cancer Res.*, **49**, 3631-3636.

MATTEI S, COLOMBO MP, MELANI C, SILVANI A, PARMIANI G AND HERLYN M. (1994). Expression of cytokine/growth factors and their receptors in human melanoma and melanocytes. *Int. J. Cancer*, **56**, 853-857.

MIGNATTI P, MORIMOTO T AND RIFKIN DB. (1991). Basic fibroblast growth factor released by single, isolated cells stimulates their migration in an autocrine manner. *Proc. Natl Acad. Sci. USA*, **88**, 11007-11011.

MINNITI CP, MAGGI M AND HELMAN LJ. (1992). Suramin inhibits the growth of human rhabdomyosarcoma by interrupting the insulin-like growth factor II autocrine growth loop. *Cancer Res.*, **52**, 1830-1835.

MINNITI CP, TSOKOS M, NEWTON WA JR AND HELMAN LJ. (1994). Specific expression of insulin-like growth factor-II in rhabdomyosarcoma tumor cells. *Am. J. Clin. Pathol.*, **101**, 198-203.

NANNI P, SCHIAFFINO S, DE GIOVANNI C, NICOLETTI G, PRODI G, DEL RE B, EUSEBI V, CECCARELLI C, SAGGIN L AND LOLLINI P-L. (1986). RMZ: a new cell line from a human alveolar rhabdomyosarcoma. *In vitro* expression of embryonic myosin. *Br. J. Cancer*, **54**, 1009-1014.

OLSON EN. (1992). Interplay between proliferation and differentiation within the myogenic lineage. *Dev. Biol.*, **154**, 261-272.

QUINN LS, EHSAN M, STEINMETZ B AND KALEKO M. (1993). Ligand-dependent inhibition of myoblast differentiation by overexpression of the type-1 insulin-like growth factor receptor. *J. Cell. Physiol.*, **156**, 453-461.

SACHSENMAIER C, RADLER-POHL A, ZINCK R, NORDHEIM A, HERRLICH P AND RAHMSDORF HJ. (1994). Involvement of growth factor receptors in the mammalian UVC response. *Cell*, **78**, 963-972.

SCHWEIGERER L, NEUFELD G, MERGIA A, ABRAHAM JA, FIDDES JC AND GOSPODAROWICZ D. (1987). Basic fibroblast growth factor in human rhabdomyosarcoma cells: implications for the proliferation and neovascularization of myoblast-derived tumors. *Proc. Natl Acad. Sci. USA*, **84**, 842-846.

SHAPIRO DN, JONES BG, SHAPIRO LH, DIAS P AND HOUGHTON PJ. (1994). Antisense-mediated reduction in insulin-like growth factor-I receptor expression suppresses the malignant phenotype of a human alveolar rhabdomyosarcoma. *J. Clin. Invest.*, **94**, 1235-1242.

STEIN CA. (1993). Suramin: a novel antineoplastic agent with multiple potential mechanisms of action. *Cancer Res.*, **53**, 2239-2248.

WAXMAN S, ROSSI GB AND TAKAKU F (EDS). (1991). *The Status of Differentiation Therapy of Cancer*, Vol. II. Serono Symposia Publications. Vol. 82. Raven Press: New York.

YUN K. (1992). A new marker for rhabdomyosarcoma. Insulin-like growth factor II. *Lab. Invest.*, **67**, 653-664.